# Recent advances in understanding the role of glucagon-like peptide 1

**DOI:** 10.12688/f1000research.20602.1

**Published:** 2020-04-06

**Authors:** Josh Reed, Stephen C. Bain, Venkateswarlu Kanamarlapudi

**Affiliations:** 1 Institute of Life Science, Medical School, Swansea University, Swansea, Wales, SA2 8PP, UK

**Keywords:** GLP-1, Type 2 diabetes, incretin, GLP-1R

## Abstract

The discovery that glucagon-like peptide 1 (GLP-1) mediates a significant proportion of the incretin effect during the postprandial period and the subsequent observation that GLP-1 bioactivity is retained in type 2 diabetes (T2D) led to new therapeutic strategies being developed for T2D treatment based on GLP-1 action. Although owing to its short half-life exogenous GLP-1 has no use therapeutically, GLP-1 mimetics, which have a much longer half-life than native GLP-1, have proven to be effective for T2D treatment since they prolong the incretin effect in patients. These GLP-1 mimetics are a desirable therapeutic option for T2D since they do not provoke hypoglycaemia or weight gain and have simple modes of administration and monitoring. Additionally, over more recent years, GLP-1 action has been found to mediate systemic physiological beneficial effects and this has high clinical relevance due to the post-diagnosis complications of T2D. Indeed, recent studies have found that certain GLP-1 analogue therapies improve the cardiovascular outcomes for people with diabetes. Furthermore, GLP-1–based therapies may enable new therapeutic strategies for diseases that can also arise independently of the clinical manifestation of T2D, such as dementia and Parkinson’s disease. GLP-1 functions by binding to its receptor (GLP-1R), which expresses mainly in pancreatic islet beta cells. A better understanding of the mechanisms and signalling pathways by which acute and chronic GLP-1R activation alleviates disease phenotypes and induces desirable physiological responses during healthy conditions will likely lead to the development of new therapeutic GLP-1 mimetic–based therapies, which improve prognosis to a greater extent than current therapies for an array of diseases.

## Introduction

Glucagon-like peptide 1 (GLP-1) was initially identified as a gut-derived incretin hormone that augments insulin secretion in a glucose-dependent manner from pancreatic islet beta cells during the postprandial period ^1, 2^. Subsequent research discovered that GLP-1 also lowered glycaemia by inhibiting glucagon secretion from pancreatic alpha cells, delaying gastric emptying and mediating induction of satiety ^1– 4^. Thus, GLP-1 maintains metabolic homeostasis during the postprandial period via multiple actions. For over a decade now, GLP-1 receptor agonists, which have a much longer half-life than endogenous GLP-1, have been an effective treatment option for type 2 diabetes (T2D) ^5, 6^. Interestingly, studies over more recent years have also identified that GLP-1 has beneficial physiological effects on a variety of tissues such as the cardiovascular and neurological systems and this has high clinical relevance given the multiple and common post-diagnosis complications associated with T2D ^1, 2, 5, 7– 10^. GLP-1 mediates its effects by binding to its receptor, the GLP-1 receptor (GLP-1R), which is a G protein–coupled receptor that is abundantly present in the pancreatic beta cells, gut, and the central nervous system (CNS) and moderately in the lung, heart, kidney, blood vessels, pancreatic alpha cells, and peripheral nervous system ^2, 4, 7^. However, studies have reported that GLP-1 exerts its effects on certain extrapancreatic tissues despite the absence of GLP-1R, which implies that this hormone may also act via currently unidentified receptors or mechanisms ^2, 7^. Therefore, given the continual emerging evidence, it has become accepted that GLP-1 has systemic physiological effects and that the actions of this hormone are not limited to mediating the incretin effect, although this is still widely accepted to be GLP-1’s most important and clinically relevant role to date ^2, 7, 11, 12^. Given these observations, GLP-1–based therapies may also provide new strategies for diseases that can arise independently of T2D in tissues susceptible to GLP-1 action ^7, 11, 13^. This review aims to summarise the well-established and speculative physiological roles of GLP-1 and highlight what studies have found over recent years in different tissues with regard to the physiological responses induced by endogenous GLP-1 and GLP-1R agonists (used for therapeutic purposes) and discuss the future implications and clinical relevance of these findings.

### GLP-1 and its mimetics: physiological roles, therapeutic uses and recent developments

Given the prevalence and incidence of T2D and the disease-associated post-diagnosis complications such as cardiovascular disease (CVD), and the observation that GLP-1 activity is retained in T2D whereas gastric inhibitory polypeptide (GIP) activity is greatly reduced, research was conducted to develop a new therapeutic strategy for T2D treatment using GLP-1–based therapies ^8– 10, 14^. Initially, success was limited, as endogenous GLP-1 and many of its early analogues have very short half-lives due to rapid inactivation by dipeptidyl peptidase 4 (DPP-4) in the circulation, requiring continuous treatment to maintain therapeutic levels, which was not practical ^6, 7, 15^. However, GLP-1 analogues were developed that had much greater half-life than endogenous GLP-1 whilst retaining the bioactivity ^12, 16^. GLP-1R agonists used in T2D treatment are either derivatives of native GLP-1 (liraglutide, albiglutide, semaglutide and dulaglutide), which have been modified to be resistant to DPP-4 inactivation, or derivatives of exendin-4 (exenatide, lixisenatide and exenatide-LR) ^4, 17^. Exendin-4, which shares 53% homology with human GLP-1, was originally isolated from the saliva of the Gila monster lizard and is resistant to the action of DPP-4 ^4^. Key pharmacological and clinical features of clinically available GLP-1R agonists are presented in Table 1.

**Table 1.** Current glucagon-like peptide 1 receptor (GLP-1R) agonists used in type 2 diabetes therapy.

GLP-1R agonist generic name (trade name)	Dosing	Half-life	Administration required before meals?
Short-acting			
Exenatide (Byetta)	Twice daily	2.4hours	Yes
Lixisenatide (Lyxumia)	Once daily	4hours	Yes
Intermediate-acting			
Liraglutide (Victoza)	Once daily	12hours	No
Long-acting			
Exenatide-LAR (Bydureon)	Once weekly	96hours	No
Albiglutide (Tanzeum) ^a^	Once weekly	6–8 days	No
Dulaglutide (Trulicity)	Once weekly	90hours	No
Semaglutide (Ozempic)	Once weekly	165–184hours	No

Information in this table is taken from 4, 16, 17.

^a^ This product was globally withdrawn in July 2018 for commercial reasons.

These agonists were found to effectively reduce hyperglycaemia in subjects with diabetes by prolonging the incretin effect and have proven to be a welcome addition to the therapeutic armamentarium in patients where insulin therapy was deemed as the next step after the failure of oral hypoglycaemic agents. GLP-1R agonist therapies have low rates of hypoglycaemia and are also associated with weight loss and not weight gain (with current dosages used in therapies), which is an undesirable side effect of some other glucose-lowering therapies ^4, 6^. The availability of various GLP-1R agonists with differing pharmacokinetic profiles enables individualised treatment options for T2D management, which has clinical relevance given patients’ differing routines, glycaemic control and diets ^4^. Furthermore, T2D is a highly multifactorial disease and pathology arises in multiple organs ^8, 9, 14^. Many of these organs express GLP-1R, which gives GLP-1 analogue therapies the potential to alleviate the diabetic phenotype systemically and the potential to provide new treatments for other diseases such as dementia and CVD in non-diabetic individuals ^2, 7^.

**Figure 1.**
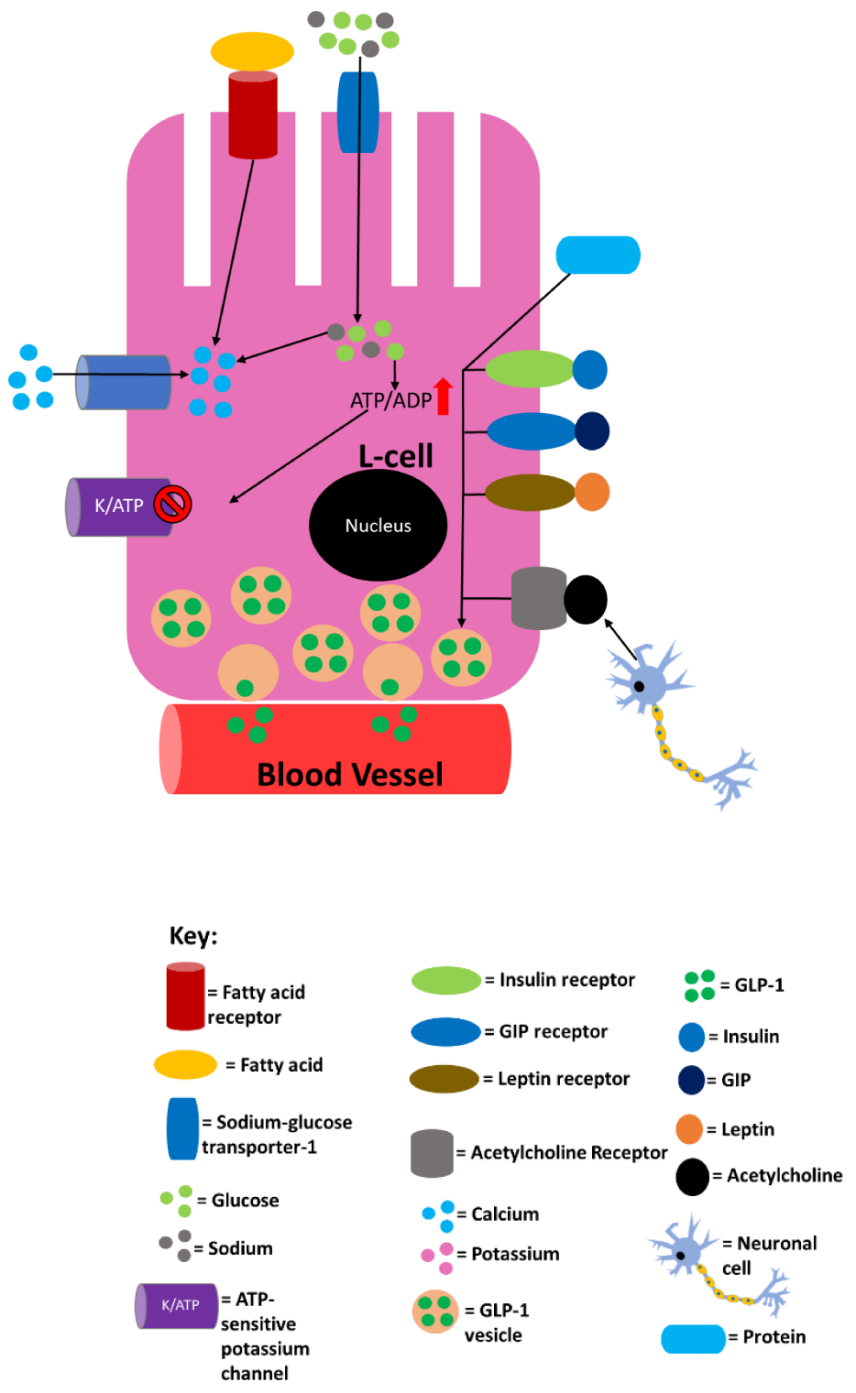
Summary of L-cell processes that are known to lead to or that may lead to glucagon-like peptide 1 (GLP-1) secretion into the circulation. Sodium entry into the L cell promotes calcium influx by inducing depolarisation, and glucose also induces calcium influx by raising ATP levels as a result of its catabolism. The binding of fatty acids to specific receptors also raises intracellular calcium levels. The now-elevated calcium levels promote exocytosis of GLP-1–containing vesicles, releasing GLP-1 into the circulation. Proteins also promote GLP-1 release, but it is not currently mechanistically understood how. L cells have been shown to express receptors for metabolic hormones, including insulin, leptin and gastric inhibitory polypeptide (GIP). The degree to which leptin and insulin stimulate overall GLP-1 secretion is still unclear, but GIP-mediated GLP-1 release has been shown to occur in rodents via acetylcholine release by the enteric nervous system. Supraphysiological concentrations of GIP potentially activate GIP receptors on L cells, potentially enhancing GLP-1 secretion. This figure and information in its legend are adapted from ^26, 37– 41^.

In the 1960s, it was shown that orally administered glucose induces a much larger insulin response than that induced by intravenously administered glucose, despite the similar resulting plasma glucose levels: the incretin effect ^18^. Subsequently, it was identified that two hormones (GIP and GLP-1) released by the gastrointestinal tract (GIT) mediate the incretin effect ^2, 7, 19^. In response to postprandial nutrient loads, enteroendocrine L cells from the intestine secrete GLP-1 ^1, 2^. It has been established that there is a direct correlation between the levels of nutrients exposed to L cells and the levels of GLP-1 in circulation ^20^. Initially, GLP-1 secretion by L cells was postulated to be dependent on postprandial glucose loads ^20, 21^. However, ingestion of mixed nutrients (carbohydrates, fats and proteins) was shown to result in greater GLP-1 secretion in comparison with just glucose ingestion ^21^. It has also been demonstrated that fats and proteins, like glucose, can independently induce GLP-1 secretion ^20^. The levels of GLP-1 in fasting plasma are about 5 to 15 pM, which increases to 40 to 60 pM during the postprandial period ^22, 23^. L cells initiate the GLP-1 response within 15 minutes after food ingestion, and GLP-1 levels peak in the circulation after about 30 minutes ^20, 24, 25^. It is still unclear how the GLP-1 response is generated so rapidly but it has been suggested that the vagal nerve and L cells in the upper jejunum part of the small intestine may be involved ^1^. However, the response is generated after the ‘cephalic phase’ of insulin secretion, implying that neuronal signals that promote insulin release do not influence GLP-1 release ^1, 23^. Rodent studies have demonstrated that, upon GIP stimulation, the nervous system promotes GLP-1 secretion from L cells, which express the receptors involved in neuronal signalling ^26, 27^. However, in chloralose-anaesthetised pigs, electrical stimulation of the vagal trunks at the level of the diaphragm had no effect on GLP-1 secretion, and human cephalic phase studies and studies in vagotomised humans found similar results ^28– 30^. Figure 1 summarises the processes in L cells that are known to lead to or that may lead to GLP-1 secretion.

Studies have revealed that the role of GLP-1 release from L cells is not limited to initiating the desired physiological response to ingested glucose but also proteins and lipids (for example, reducing triglyceride excursions), giving GLP-1 a more diverse role in maintaining metabolic homeostasis during the postprandial period ^2, 20, 21^. Additionally, understanding the neuronal regulation of GLP-1 release in humans is unclear, as is how other hormones involved in metabolic homeostasis regulate L-cell GLP-1 secretion ^26, 27^.

GLP-1 has a very short half-life (1–2 minutes) in the circulation ^1^ because of its rapid inactivation by proteolytic actions of DPP-4 ^1, 7^. Consequently, it has been become generally accepted that about 85% of GLP-1 exists in the inactive form in circulation ^23^. Since the majority of GLP-1 is inactivated rapidly after secretion, its response is highly inefficient with regard to ATP expenditure (GLP-1 synthesis requires ATP) ^2, 23^. It has become generally accepted that endogenous GLP-1 activates sensory afferents of the enteric nervous system, which helps to promote the desired postprandial response, in addition to mediating the incretin effect via the endocrine route to islet beta cells ^1, 2, 31, 32^. Although it is still unclear why the GLP-1 response is so wasteful from a bioenergetics perspective, recent studies have suggested that the inactive GLP-1 has actions on the liver, vasculature and heart which are comparable to those of insulin ^33^. However, the notion that GLP-1 has any action on the liver is an area of controversy, given the absence of GLP-1R in hepatocytes ^2^. Therefore, it is possible that the ‘inactive forms’ are not actually inactive and act on currently unidentified signalling pathways and this seems likely to be the case as it is very unlikely that evolution has developed such an ATP-wasteful response. However, GLP-1R agonists (such as liraglutide and exenatide) that are used in T2D therapy are all synthetically developed. In contrast to endogenous GLP-1, they have a half-life of several hours or more as they are resistant to DPP-4 inactivation ^4, 34^. This results in prolonged pancreatic beta-cell GLP-1R activation that in turn prolongs the incretin effect, resulting in a reduction of hyperglycaemia in most patients ^4, 35^.

**Figure 2.**
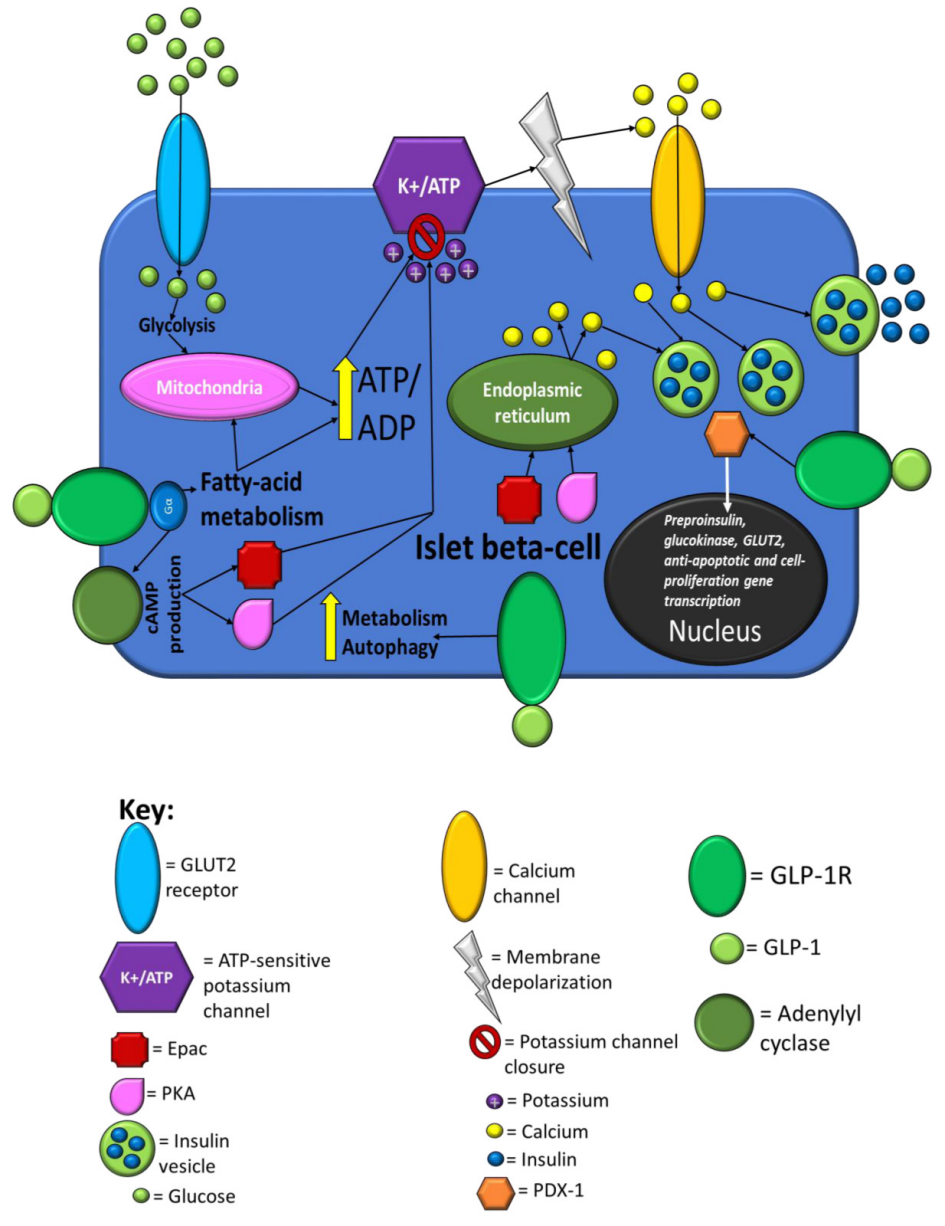
Summary of islet beta-cell processes that are influenced by postprandial glucose loads and glucagon-like peptide 1 (GLP-1) action. When the plasma glucose rises, glucokinase initiates glucose catabolism, resulting in an increase in the cell’s ATP levels, which results in closure of ATP-sensitive potassium channels, causing membrane depolarisation and calcium influx. The resulting calcium influx triggers insulin secretion by promoting movement of insulin vesicles to the cell membrane. The binding of GLP-1 to GLP-1R enhances glucose-stimulated insulin secretion by initiating cAMP production via adenylyl cyclase activity that in turn activates protein kinase A (PKA) and exchange protein directly activated by cAMP (Epac). Activated PKA and Epac augment extracellular calcium influx induced by glucose catabolism via further promoting closure of potassium channels, and Epac also raises the intracellular calcium levels directly via promoting calcium release from the endoplasmic reticulum. Evidence suggests that PKA increases the permeability of calcium channels, which further enhances the influx of extracellular calcium. The elevated intracellular calcium levels further enhance exocytosis of insulin vesicles. The activation of GLP-1R also induces activation of PDX-1 transcription factor and its translocation to the nucleus, which results in the transcription of the preproinsulin, GLUT2 and glucokinase genes for further insulin production, glucose uptake and glucose catabolism, respectively. PDX-1 activation also induces the transcription of genes involved in apoptotic resistance, neogenesis and proliferation, giving GLP-1 a role in promoting beta-cell survival during the postprandial period in addition to enhancing insulin secretion. Studies have found that GLP-1R activation enhances autophagy and metabolic profiles and reduces ER stress via PKA-dependent mechanisms. This figure and information in its legend are adapted from ^1, 2, 46– 52, 56^.

The incretin hormones play a crucial role in maintaining metabolic homeostasis since about 60 to 70% of the total postprandial insulin released into circulation is due to the action of these hormones ^2, 36^. GIP and GLP-1 account for about 60% and 40% of the incretin effect, respectively ^2^. The action of GLP-1 is dependent on blood glucose levels since it can only potentiate glucose-stimulated insulin secretion from islet beta cells ^7^. Furthermore, GLP-1 suppresses glucagon secretion from islet alpha cells, but only when glucose levels are above fasting, which assists in the ability of postprandial insulin to mediate anabolism of ingested nutrients ^1, 2, 7^. Given the importance of the correct balance of insulin and glucagon activity being maintained to mediate desired net systemic anabolic or catabolic metabolic effects in response to nutrient supply and demand, GLP-1 activity plays a critical role in maintaining metabolic homeostasis during the postprandial period via insulinotropic and glucagonostatic effects ^2, 4^. Activation of GLP-1R results in a rapid (seconds to minutes) potentiation of insulin secretion from islet beta cells which is achieved via rapid cAMP production, which results in the activation of protein kinase A (PKA) and exchange protein directly activated by cAMP (Epac), and these two effectors modify several targets in the secretory machinery, resulting in enhanced insulin secretion ^2, 12^. Additionally, GLP-1R activation results in transcription of genes involved in proliferation, neogenesis and apoptotic resistance in islet beta cells, expanding GLP-1’s role from potentiating insulin secretion from islet beta cells to promoting their survival via translocation of pancreatic duodenal homeobox 1 (PDX-1) transcription factor to the nucleus ^2, 12, 42^. Chronic liraglutide treatment in diabetic mice was shown to prevent loss of beta-cell mass, by increasing the proliferation of beta cells and decreasing beta-cell apoptosis, after alloxan injection ^35^. Inhibition of beta-cell apoptosis in isolated human pancreatic beta cells was also reported after liraglutide administration, and the beta-cell proliferation rate was increased up to threefold after incubation for 24 hours ^43^. GLP-1/exendin-4 has also been shown to facilitate beta-cell neogenesis in rat and human pancreatic ducts ^44^. Endogenous GLP-1 not only protects against apoptosis but also induces proliferation of rat primary islet cells and beta-cell lines ^12, 45^. GLP-1R activation has also been proposed to reduce lipotoxicity, glucotoxicity, Ca ^2+^ depletion, excess nitric oxide, cytokine-induced endoplasmic reticulum (ER) stress and oxidative stress in both primary beta cells and cell lines via multiple downstream signalling pathways ^46– 50^. One study found that GLP-1R signalling alters the intracellular response from the translational repression to the translational recovery phase in a PKA-dependent manner ^46^. GLP-1 has recently been shown to regulate autophagy in beta cells ^51, 52^. Exendin-4 treatment was shown to facilitate autophagy in rat INS-1E cells and isolated human islets under chronic exposure to excess nutrients by preventing autophagosomal-lysosomal fusion impairment. Its treatment was also reported to increase lysosomal function, which improved autophagosome clearance and thereby reduced islet beta-cell injury in a rat model of tacrolimus-induced diabetes ^53^. Additionally, exendin-4 treatment *in vivo* has been shown to decrease tacrolimus-induced oxidative stress, hyperglycemia, and apoptosis [43]. Interestingly, recent studies have found that chronic GLP-1R activation results in metabolic reprogramming associated with upregulation of glycolytic enzymes and increased ATP production ^54^. It has been postulated that an enhancement in metabolism by GLP-1 treatment likely decreases ER stress by increasing intracellular Ca ^2+^ and mitochondrial ATP levels, which then are used in the maintenance of ER homeostasis ^12^. Interestingly, chronic GLP-1R activation also results in the activation of distinct signalling pathways; for instance, GLP-1R agonists have been reported to promote the secretion of insulin-like growth factor 2 and induce expression of its receptor, which is thought to contribute to the pro-survival abilities of GLP-1 in islet beta cells ^12, 55, 56^. Given that ER stress, impaired autophagy and proliferation, and increased apoptosis are all thought to contribute to islet beta-cell dysfunction in T2D, and the findings that GLP-1R activation can alleviate all of these, suggests GLP-1 and its analogues do more than just augmenting insulin secretion ^12^. However, currently, there are no data from recent long-term clinical studies to suggest that GLP-1–based therapies exert protective effects on islet beta cells, and it is unclear whether the maximum insulin enhancement is achieved with present dosages used clinically, which are currently limited by side effects ^10, 57– 60^. Figure 2 summarises the processes in islet beta cells that are influenced by postprandial glucose loads and GLP-1 action.

The regulation of alpha cells by GLP-1 remains an area of controversy ^2, 7, 61, 62^. However, it is firmly established that the ability of GLP-1 to inhibit glucagon secretion is not dependent on its insulinotropic effects, given that GLP-1 is able to lower fasting plasma glucose levels in people with type 1 diabetes ^7, 63^. Despite the low and controversial GLP-1R expression in islet alpha cells, recent studies have provided evidence that GLP-1R agonists mediate direct effects on alpha cells via the presence of GLP-1R ^7, 61, 62^. Islet alpha-cell GLP-1R knockout mice failed to inhibit glucagon secretion at high glucose levels; interestingly, these mice had impaired glucagon secretion during low glucose conditions ^62^. Another recent study found that GLP-1 action on human pancreatic islet alpha cells was not dependent on paracrine signalling since preventing insulin and somatostatin signalling had no effect on the inhibition of glucagon secretion ^61^. Until recently, there was a strong consensus in the literature that GLP-1 acts on alpha cells through an unidentified receptor or through paracrine mechanisms (or both) via insulin and somatostatin release from islet beta and delta cells, respectively ^2, 7^. Hyperglycaemia in T2D is now accepted to be induced by both hyperglucagonemia and hypoinsulinemia, and up to 50% of the excess plasma glucose levels in patients has been postulated to arise from inappropriate glucagon secretion ^64, 65^. Given that T2D is now considered to be a bihormonal disorder, how GLP-1R agonists influence glucagon secretion is of high clinical relevance ^7, 66^.

**Figure 3.**
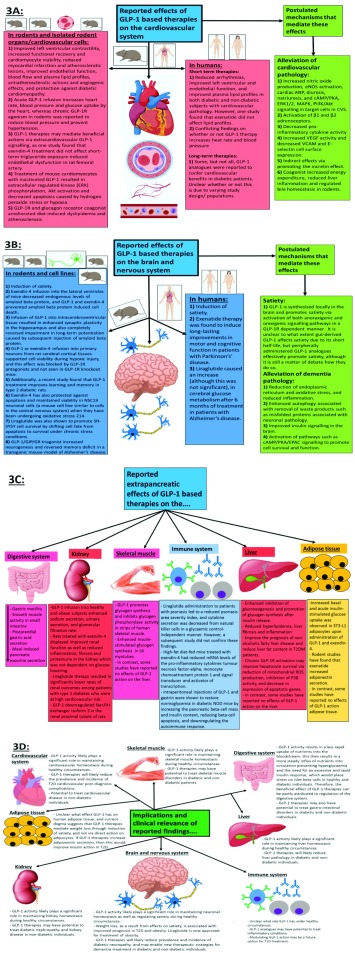
Summary of the reported extrapancreatic effects of glucagon-like peptide 1 (GLP-1)-based therapies (A–C) and the clinical implications of these findings (D). In frame C, organs that are underlined indicate that GLP-1 receptor (GLP-1R) expression is controversial/unconfirmed, and it is not clear whether or how GLP-1 is able to mediate any direct physiological effects on them. ANP, atrial natriuretic peptide; CVS, cardiovascular system; eNOS, endothelial nitric oxide synthase; EPAC, exchange protein directly activated by cAMP; ERK1/2, extracellular signal-related kinase 1/2; MAPK, mitogen-activated protein kinase; NOD, non-obese diabetic; PI3K, phosphoinositide-3-kinase; PKA, protein kinase A; ROS, reactive oxidative species; T1D, type 1 diabetes; T2D, type 2 diabetes; T2DM, type 2 diabetes mellitus; VCAM, vascular cell adhesion molecule-1; VEGF, vascular endothelial growth factor. This figure and information in its legend are adapted from 1, 2, 7, 12, 77– 98.

Studies have revealed that GLP-1 has multiple extrapancreatic targets and effects, which not only assists with inducing the desired physiological response during the postprandial period but also has beneficial effects on alleviating pathology associated with certain tissues and organs systemically, which arises because of T2D manifestation or independently of T2D ^2, 8, 9, 12^. These findings are of clinical importance since there is demand for new therapeutic strategies that both reduce the prevalence and incidence of T2D post-diagnosis complications and provide better prognosis than current treatments for diseases such as dementia ^2, 7– 9, 12^. Indeed, recent studies have found that certain GLP-1 analogue therapies confer cardiovascular benefits to people with diabetes, which has high clinical importance given the prevalence of the cardiovascular comorbidity of diabetes ^10^. Owing to the weight loss achieved as a result of the ability of this drug to mediate satiety, liraglutide was also recently approved for the treatment of obesity in individuals without diabetes ^67^. It was initially postulated that the effect of GLP-1 on appetite could be due to its negative regulation of gut motility; however, evidence that GLP-1 has direct effects on specific neurons in the hypothalamus has emerged ^24^. GLP-1 is expressed in neurons of the brainstem and GLP-1R is present in the hypothalamic areas that control energy homeostasis and food intake, including the arcuate nucleus, paraventricular nucleus, and dorsomedial nucleus ^1, 68^. Intracerebroventricular injection of GLP-1 inhibits food intake in rats and this activity is blocked by exendin _9–39_, demonstrating that GLP-1 has direct effects on neurons ^69, 70^. Studies have shown that intracerebroventricular administration of GLP-1 in rodents induces satiety even in the absence of food in the GIT and when gastric emptying has been inhibited; thus, GLP-1 induces satiety via its direct effects on neurons in the caudal brainstem ^1, 24, 68^. The mechanism by which peripherally administered GLP-1 induces satiety has yet to be elucidated, but it likely involves signals being generated by GLP-1 binding to GLP-1R on neurons in the GIT, hepatoportal bed and CNS ^1, 71^. The differences in weight loss reported for the various GLP-1 analogues currently used to treat T2D are likely related to their penetration of the CNS, allowing central GLP-1R binding ^72, 73^. Additionally, some studies have found evidence that GLP-1–based therapies alleviate pathology associated with other diseases such as dementia in non-diabetic individuals, although there is controversy about the potential for these therapies in dementia treatment based on different findings, especially in human studies ^12, 74^. Non-alcoholic fatty liver disease (NAFLD) and subsequent non-alcoholic steatohepatitis (NASH) are highly prevalent among individuals with T2D or obesity (or both) because of the excessive hepatic fat deposition associated with these diseases ^75, 76^. Studies have found that GLP-1R agonist treatments reduce liver pathology not only by promoting weight loss and improving glycaemic control but also by reducing alanine aminotransferase (ALT) levels, which was linked to the degree of weight loss induced ^75^. The ability of GLP-1R agonists to reduce weight and lower ALT levels suggests a role for these compounds in treating NAFLD/NASH and reducing liver damage. A recent study found that semaglutide treatment significantly reduced ALT levels in NAFLD at-risk patients, and histological data are awaited from an ongoing phase 2 trial of semaglutide in biopsy-proven NASH ^76^. Figure 3 summarises the findings from studies on the extrapancreatic effects of GLP-1-based therapies and the clinical implications of these findings.

A recent longitudinal study found that GLP-1–based agonist action may not only be an effective treatment for T2D but also prevent or delay disease manifestation in at-risk individuals: Liraglutide treatment reduced the risk of T2D manifestation in obese individuals and individuals who had a body mass index of more than 27 and had hypertension or dyslipidaemia; after 160 weeks, the liraglutide-treated group (n = 1472) had a smaller percentage of the population with diagnosed T2D (2% versus 6%) than the placebo-treated control group (n = 738) ^99^. Additionally, time to T2D onset during the 160-week study was found to be 2.7 times longer in the liraglutide-treated group than the placebo control group. Interestingly, recent studies have also demonstrated that the therapeutic potential of GLP-1 action in the treatment of diseases such as T2D and dementia can likely be enhanced by co-activation of other receptors involved in maintaining metabolic homeostasis. The dual GLP-1R and GIP receptor (GIPR) agonist treatment induced better glycaemic control and body weight reduction in diet-induced obesity (DIO) mice compared with liraglutide (GLP-1R agonist)-only-treated controls ^100^. Co-agonism of GLP-1R and glucagon receptor (GR) was also shown to reduce body weight synergistically in rodent obesity models ^101^. These co-agonists have also improved glycaemic control in monkeys and humans ^100^. In a recent study, tirzepatide, a novel GLP-1R and GIPR co-agonist, was found to induce greater glucose control and weight loss in human patients with T2D than duglatide (a GLP-1R–only agonist) ^102^. Recently, a triple agonist for GLP-1R, GR and GIPR was developed and tested on DIO mice, and it was found that the tri-agonist lowered body weight to a greater extent than GLP-1R/GIPR co-agonist treatment ^103^. The co-agonist and the tri-agonist were equally effective at reducing blood glucose levels and improving glucose tolerance without the induction of hypoglycaemia and this demonstrates that chronic GR agonism does not counteract the anti-hyperglycemic effects of GLP-1R and GIPR activity. Surprisingly, tri-agonist treatment lowered plasma insulin levels to a greater extent than the GLP-1R/GIPR co-agonist, indicating improved insulin sensitivity. Interestingly, no difference in food intake was observed between wild-type mice treated with the dual incretin GLP-1R/GIPR co-agonist and those treated with the tri-agonist despite the difference in weight loss and this was found to be due to significantly enhanced ATP expenditure in tri-agonist–treated DIO mice. The capacity of the tri-agonist to prevent the development of spontaneous diabetes compared with the dual incretin GLP-1R/GIPR co-agonist was also tested in mouse models of T2D. The tri-agonist treatment prevented the excessive weight gain in vehicle-treated mice to a greater degree than the GLP-1R/GIPR co-agonist and this difference was not due to cumulative food intake. The tri-agonist also protected these mice from fasting hyperglycaemia and to a better degree than the GLP-1R/GIPR co-agonist. Interestingly, the tri-agonist also significantly reduced alpha-cell infiltration into the core of pancreatic islets, helping to preserve the islet architecture observed in healthy pancreatic islets. Glycaemic improvements were maintained in Zucker diabetic fatty rats 3 weeks after treatment cessation although they had gained body weight and were comparable in mass to vehicle-treated controls, demonstrating that the tri-agonist delays T2D progression in rodent models of spontaneous diabetes. Interestingly, this study also demonstrated that the effects of the tri-agonist are dependent on an excess of nutrient storage as weight and food intake were not altered in lean mice even after chronic treatment with the tri-agonist. A recent study also found that tri-agonist treatment was able to ameliorate diet-induced steatohepatitis in mice ^77^. However, the clinical importance of the co- and tri-agonists has yet to be established since the majority of the studies using these agonists were carried out using rodent models. Some GLP1R/GIPR co-agonists are in clinical development for T2DM treatment.

### Summary

In summary, abundant evidence has emerged over recent years to demonstrate that GLP-1 has multiple pancreatic effects and extrapancreatic targets and actions throughout the body, which likely play a significant role in maintaining metabolic homeostasis during healthy conditions and therefore to label GLP-1 as just an ‘incretin hormone’ is now outdated ^1, 2, 7, 12^. Thus, GLP-1 has a much more complex and diverse physiological role than previously thought. Despite all the research that has been conducted on GLP-1 and its actions, the mechanisms that regulate the secretion of this hormone are still not fully understood, nor are the mechanisms by which it exerts its actions. It is unclear whether GLP-1 is able to mediate its effects by an unidentified receptor, and there is controversy as to whether the inactive forms are able to induce physiological effects ^2, 7, 12^. Native GLP-1 has no use therapeutically, but the advent of GLP-1R agonists that have much longer half-lives has enabled GLP-1–based actions for therapeutic uses as a viable option ^5– 7^). Initially, GLP-1R agonists were thought to be limited to T2D treatment, but it has become clear that these drugs have the potential to treat other diseases such as obesity, CVD, dementia and NAFLD ^2, 7, 12, 77^, 103. Better understanding the mechanisms and signalling pathways by which acute and chronic GLP-1R activation both alleviates disease phenotypes and induces desirable physiological responses during healthy conditions will likely lead to the development of new therapeutic GLP-1 action–based therapies, which improve prognosis to a greater extent than current therapies. Importantly, the observations that GLP-1–based therapies delay/prevent the manifestation of T2D ^99^ may enable expansion of the role of GLP-1 mimetics from treating diseases to delaying or preventing diseases manifesting in tissues susceptible to GLP-1–based action. Additionally, given the observations from co- and tri-agonist studies, future research will need to investigate what the long-term effects of synergistic activation of other receptors will be ^78^, 100, 101, 103. Finally, over recent years, allosteric agonists that act on sites distinct from those of GLP-1 and its mimetics have been developed for the GLP-1R ^23^. Research is ongoing to determine whether these agonists can modulate GLP-1R downstream effectors to better alleviate pathology than current GLP-1R agonists used in the therapy. One advantage of these allosteric agonists is that, in contrast to current therapies which require injection, they can be administered orally. Ongoing and future research into the modulation of GLP-1 action will likely lead to the development of new therapeutic strategies for an array of diseases.

## Abbreviations

ALT, alanine aminotransferase; CNS, central nervous system; CVD, cardiovascular disease; DIO, diet-induced obesity; DPP-4, dipeptidyl peptidase 4; ER, endoplasmic reticulum; GIP, gastric inhibitory polypeptide; GIPR, gastric inhibitory polypeptide receptor; GIT, gastrointestinal tract; GLP-1, glucagon-like peptide 1; GLP-1R; glucagon-like peptide 1 receptor; GR, glucagon receptor; NAFLD, non-alcoholic fatty liver disease; NASH, non-alcoholic steatohepatitis; PKA, protein kinase A; T2D, type 2 diabetes
